# The molecular cytogenetic characterization of *Conopophaga lineata* indicates a common chromosome rearrangement in the Parvorder Furnariida (Aves, Passeriformes)

**DOI:** 10.1590/1678-4685-GMB-2020-0018

**Published:** 2020-06-12

**Authors:** Thays Duarte de Oliveira, Rafael Kretschmer, Natasha Ávila Bertocchi, Patricia C.M. O’Brien, Malcolm A. Ferguson-Smith, Analía del Valle Garnero, Edivaldo Herculano Correa de Oliveira, Ricardo José Gunski

**Affiliations:** 1 Universidade Federal do Rio Grande do Sul (UFRGS), Programa de Pós-Graduação em Biologia Animal, Porto Alegre, RS, Brazil.; 2 Universidade Federal do Rio Grande do Sul (UFRGS), Programa de Pós-Graduação em Genética e Biologia Molecular, Porto Alegre, RS, Brazil.; 3 University of Cambridge, Department of Veterinary Medicine, Cambridge, United Kingdom.; 4 Universidade Federal do Pampa (UNIPAMPA), Programa de Pós-Graduação em Ciências Biológicas, São Gabriel, RS, Brazil.; 5 Instituto Evandro Chagas, Seção Meio Ambiente (SAMAM), Ananindeua, PA, Brazil.; 6 Universidade Federal do Pará, Belém, Instituto de Ciências Exatas e Naturais, Belém, PA, Brazil.

**Keywords:** Birds, avian chromosomal evolution, chromosomes, rDNA

## Abstract

Cytogenetic analyses of the Suboscines species are still scarce, and so far, there is no karyotype description of any species belonging to the family Conopophagidae. Thus, the aim of this study is to describe and analyze the karyotype of *Conopophaga lineata* by chromosome painting using *Gallus gallus* (GGA) probes and to identify the location of the 18/28S rDNA cluster. Metaphases were obtained from fibroblast culture from two individuals of *C. lineata*. We observed a diploid number of 2n=78. GGA probes showed that most ancestral syntenies are conserved, except for the fission of GGA1 and GGA2, into two distinct pairs each. We identified the location of 18S rDNA genes in a pair of microchromosomes. The fission of the syntenic group corresponding to GGA2 was observed in other Furnariida, and hence may correspond to a chromosomal synapomorphy for the species of Parvorder Furnariida.

The order Passeriformes encompasses approximately 5700 species, equivalent to 60% of existing birds, and besides their large diversity, they can also be considered a cosmopolitan group, thus becoming the focus of different studies. The order encompasses two large Suborders - Oscines and Suboscines (Ericson *et al.*, 2003, 2014). The process of song learning is the main distinction between Oscines and Suboscines: Oscines are characterized by complex vocalizations, which are often learned through imitation. On the other hand, Suboscines have less complex vocal organs, and their songs do not seem to be learned by imitation (Raikow and Bledsoe, 2000). Suboscines are traditionally divided into two infraorders - Tyrannides (272 genera), endemic to the New World, and Eurylaimides (12 genera), which are widely distributed in the Old World (Selvatti *et al.*, 2015). Tyrannides are divided into the Parvorders - Furnariida and Tyrannida (Selvatti *et al.*, 2015). The species *Conopophaga lineata* (CLI), the focus of this study, belongs to the Conopophagidae family, which is included in the Parvorder Furnariida.

Most cytogenetic studies in Passeriformes have used classical approaches and, among birds, this is the order with the largest number of species analyzed (Kretschmer *et al.*, 2018a). Of the twenty species of this order examined by molecular genetics so far, six belong to the Suborder Suboscines: *Elaenia spectabilis, Pitangus sulphuratus, Serpophaga subcristata* and *Satrapa icterophrys* (Tyrannida - Tyrannidae), *Synallaxis frontalis* and *Glyphorynchus spirurus* (Furnariida - Furnariidae) (Guttenbach *et al.*, 2003; Derjusheva *et al.*, 2004; Itoh and Arnold, 2005; Kretschmer *et al.*, 2015; Santos *et al.*, 2017; Rodrigues *et al.*, 2017; Kretschmer *et al.*, 2018b; Ribas *et al.*, 2018). Using as reference the putative ancestral karyotype of birds (Griffin *et al.*, 2007), all species analyzed by chromosome painting with *Gallus gallus* probes (GGA) have presented conservation of ancestral macrochromosomes, except for ancestral pair 1- which corresponds to two pairs representing a synapomorphy for Passeriformes – and pair 2, which has undergone fission in *Satrapa icterophrys* (Parvorder Tyrannida), *Synallaxis frontalis* and *Glyphorynchus spirurus* (Parvorder Furnariida) (Rodrigues *et al.*, 2017; Kretschmer *et al.*, 2018b; Ribas *et al.*, 2018). Additionally, the use of 18S rDNA probes has revealed that the number and distribution of NORs varies from 1-3 pairs in Passeriformes (Kretschmer *et al.*, 2014, 2015; Rodrigues *et al.*, 2017).

Despite these data, information on events occurring during the karyotype evolution of Passeriformes is still fragmentary, as observed in most groups of birds. In this sense, studies involving species from basal clades are important to reconstruct the sequence of rearrangements arising during Passeriformes diversification. Considering that Conopophagidae represents one of the most basal lineages of passerines (Selvatti *et al.*, 2015), a detailed study of one species of this family may shed some light on the chromosome evolution of Passeriformes. Hence, we describe here for the first time the karyotype of a species belonging to this family, the rufous gnateater (*Conopophaga lineata*).

The protocols were approved by the Committee of Ethics on the use of Animals (CEUA- Universidade Federal do Pampa, 026/2012), and SISBIO (Permission Number: 101 33860-4). Skin biopsies were collected from two females of *C. lineata* in Porto Vera Cruz and São Gabriel (Rio Grande do Sul, Brazil), and used for cell culture, following Sasaki *et al.* (1968), with modifications. In this process, cells were dissociated with collagenase type IV (Sigma) and grown in DMEM medium supplemented with fetal bovine serum (20%). Chromosome preparations were obtained after exposure to colcemid (1 h, 37 ºC), hypotonic treatment (0.075M KCl, 15 min, 37 ºC) and methanol/acetic acid (3:1). fixative

Fluorescence *in situ* hybridization (FISH) experiments were performed using whole chromosome probes from *Gallus gallus* (GGA 1-10), obtained by flow cytometry at the Cambridge Resource Centre for Comparative Cytogenetics, (Cambridge, UK), amplified and labeled with biotin by DOP-PCR. Hybridizations were carried out according to Oliveira *et al.* (2010). Detection was performed with the use of Streptavidin-CY3 (Invitrogen). 18S rDNA probe fragments were labeled with digoxigenin by Nick Translation (Nick Translation Kit, Roche) and detected with Anti-Digoxigenin-Rhodamine, following the manufacturer's instructions, slide preparation, hybridization and washing were performed according to Daniels and Delany (2003).

Approximately 30 mitotic metaphases from each specimen were analyzed in order to determine the diploid number, chromosome morphology and confirm FISH experiments. Metaphases were analyzed in an epifluorescence light microscope (Imager Z2, Zeiss, Germany), and the images were acquired with the software Axiovision 4.8 (Zeiss, Germany).

The diploid number of *C. lineata* is 78. Pairs 1 to 7 are acrocentric, except for pair 4, which is submetacentric. The other autosomal chromosomes are telocentric, while the Z sex chromosome is submetacentric and W sex chromosome possibly is a telocentric microchromosome (Figure 1).

**Figure 1:**
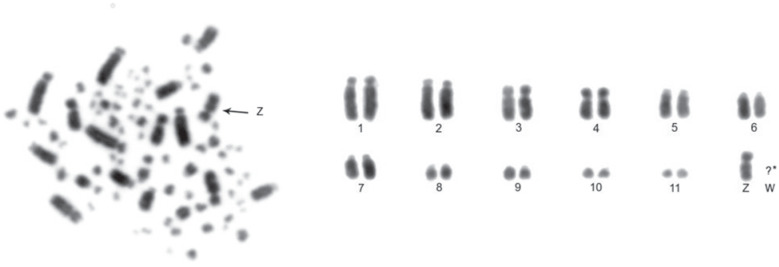
Metaphase and partial karyotype of a female specimen of *Conopophaga lineata.* *It was not possible to identify the W sex chromosome.

GGA probes 1-10 produced 13 different signals, revealing chromosome rearrangements. Most of the ancestral macrochromosomes are conserved in *C. lineata*, except for GGA1 and GGA2, which are fissioned in two pairs each. GGA 4 probe hybridized to two chromosome pairs, as in the putative bird ancestral karyotype. GGA3 and 5-10 hybridized to only a single pair each, revealing conserved syntenies. In addition, CLI 5 is the result of a fusion between a segment of GGA2 and an unidentified chromosome, possibly a microchromosome (Figures 2A, B and 3).

**Figure 2:**
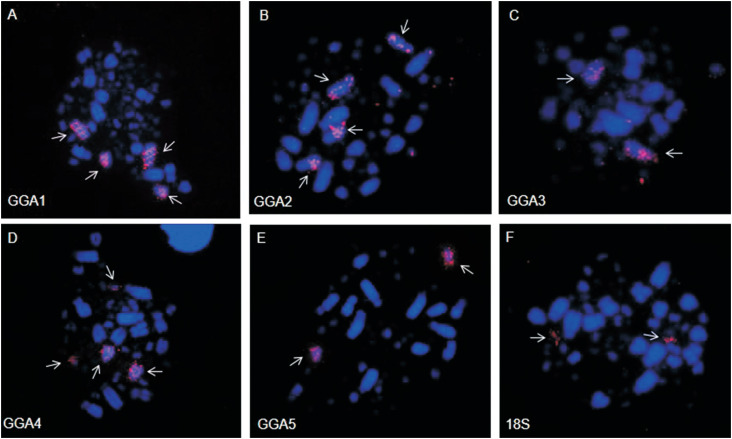
Representative FISH experiments with GGA1 (A), GGA2 (B), GGA3 (C), GGA4 (D), GGA5 (E) and 18S rDNA probes (F) in metaphases of *Conopophaga lineata*. Arrows indicate the homologous chromosomes to the probes used.

**Figure 3:**
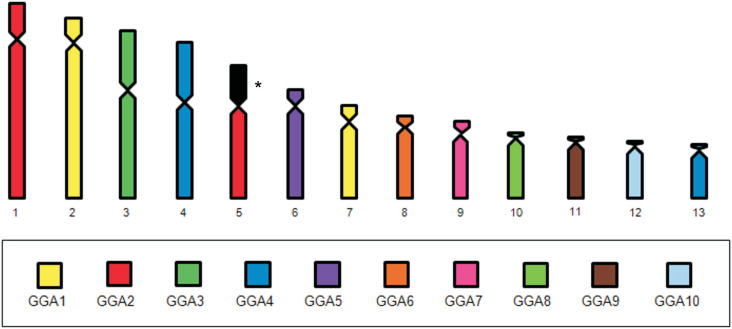
Homology map of *Conopophaga lineata* with *Gallus gallus* (GGA) probes indicated by color. *Not hybridized segment with any GGA probes used.

The diploid number observed, 2n = 78, is found in most bird species and is similar to the hypothetical bird ancestor (80 chromosomes) (Griffin *et al.*, 2007). It was possible to observe that the first and second pairs have a similar size, differently from most of Passerines studied so far (Kretschmer *et al.*, 2014; Santos *et al.*, 2017), indicating an additional fission in *Conopophaga lineata*.

In fact, FISH results (Figure 2) revealed that GGA 1, 2 and 4 probes hybridized on two chromosome pairs each, whereas all other probes hybridized to only one chromosome pair each. While GGA1 fission is commonly found in Passeriformes and considered a synapomorphy for this group (Kretschmer *et al.*, 2015; Santos *et al.*, 2017), the hybridization of GGA4 to two chromosome pairs - CLI4 and CLI13 (Figure 2D) - is common to most birds, representing the ancestral state, and hence, in *G. gallus* this pair is the result of the fusion of two chromosomes of the putative avian ancestral karyotype (PAK), PAK4 and PAK10 (Griffin *et al.*, 2007, Kretschmer *et al.*, 2018a). Additionally, centric fission of GGA1 is also observed in species of the orders Strigiformes, Psittaciformes, Falconiformes, and Accipitriformes (Guttenbach *et al.*, 2003; Oliveira *et al.*, 2005, 2008; Nanda *et al.*, 2006, 2007).

Interestingly, the fission of GGA2, into two chromosomes in *C. lineata* (CLI1 and CLI5q) (Figure 3), is atypical for Passeriformes; normally GGA2 is conserved and corresponds to the largest pair (Table 1) (Kretschmer *et al.*, 2014, 2015; Santos *et al.*, 2017). Moreover, the centric fission of GGA2 was observed in other Suboscines species, belonging to parvorder Furnariida - *Synallaxis frontalis* (Kretschmer *et al.*, 2018b) and *Glyphorynchus spirurus* (Ribas *et al.*, 2018) -, and parvorder Tyrannida - *Satrapa icterophrys* (Rodrigues *et al.*, 2017), which also shows pairs 1 and 2 with similar sizes, as in *C. lineata*. Hence, this fission explains the minimum size difference between the first and second pairs in other Suboscines species in which only classical cytogenetic data (Giemsa staining and chromosome banding) are available, such as *Sittasomus griseicapillus, Lepidocolaptes angustirostris* (Dendrocolaptidae) and *Pyriglena leucoptera, Dysithamnus mentalis* (Formicariidae) – all of them are members of Parvorder Furnariida (Ledesma *et al.*, 2002; Moyle *et al.*, 2009; Barbosa *et al.*, 2013; Kretschmer *et al.*, 2018b). Consequently, GGA2 fission in species of parvorder Furnariida and in *Satrapa icterophrys* of parvorder Tyrannida may be indicative of convergent evolution (Table 1).

**Table 1:** Rearrangements in putative avian ancestral karyotype homologous segments (PAK1-10) in Suboscines species.

Parvorders	Species	Rearrangements	References
Tyrannida	*Elaenia spectabilis*	fission PAK1 (ESP2 and 5)	Kretschmer *et al.*, 2015
Tyrannida	*Pitangus sulphuratus*	fission PAK1 (PSU3 and 5)	Rodrigues *et al.*, 2018
Tyrannida	*Serpophaga subcristata*	fission PAK1 (SSU3 and 5)	Rodrigues *et al.*, 2018
Tyrannida	*Satrapa icterophrys*	fission PAK1 (SIC2 and 5) fission PAK2 (SIC3 and 7)	Rodrigues *et al.*, 2018
Furnariida	*Synallaxis frontalis*	fission PAK1 (SFR1 and 5) fission PAK2 (SFR3 and 7)	Kretschmer *et al.*, 2018b
Furnariida	*Glyphorynchus spirurus*	fission PAK1 (GSP3 and 4) fission PAK2 (GSP2 and 5)	Ribas *et al.*, 2018
Furnariida	*Conopophaga lineata*	fission PAK1 (CLI2 and 7) fission PAK2 (CLI1 and 5q)	Present study

In addition to the fission of GGA2, we have identified that pair 5 of *C. lineata* was formed from a fusion between one of the segments originated from the GGA2 fission and a microchromosome (Figure 3).

Despite the fact that these rearrangements have been observed in a species belonging to the basal family Conopophagidae, the localization of ribosomal clusters in a pair of microchromosomes, corresponds to a plesiomorphic characteristic, usually observed in the order Passeriformes and in other avian orders, demonstrating the conservation of the ancestral state (Figure 2F) (Nishida-Umehara *et al.*, 2007; Oliveira *et al.*, 2017; Santos *et al.*, 2017).

In conclusion, we demonstrate that the morphology of macrochromosomes in *C. lineata* is significantly different from other Passeriformes species. Furthermore, we found a fission in GGA2, which appears to be a common chromosome rearrangement in Furnariidae and possibly other Parvorder Furnariida species that have minimal size difference between the first chromosomal pairs, in addition to the fissions that are typically found in Passeriformes (GGA1). However, since passerines present a high degree of chromosomal rearrangement, subsequent mapping and sequencing studies allowing the investigation of intrachromosomal rearrangements may elucidate these events.
